# Comparison of resistive capacitive energy transfer therapy on cadaveric molars and incisors with and without implants

**DOI:** 10.1038/s41598-022-16189-0

**Published:** 2022-07-13

**Authors:** Albert Pérez-Bellmunt, Jordi Caballé-Serrano, Jacobo Rodríguez-Sanz, César Hidalgo-García, Vanessa González-Rueda, Sergi Gassó-Villarejo, Daniel Zegarra-Chávez, Carlos López-de-Celis

**Affiliations:** 1 Faculty of Medicine and Health Sciences, Universitat Internacional de Catalunya, Campus Sant Cugat, Carrer de Josep Trueta s/n, Sant Cugat del Vallès, 08195 Barcelona, Spain; 2 ACTIUM Anatomy Group, Carrer de Josep Trueta, Sant Cugat del Vallès, 08195 Barcelona, Spain; 3 Department of Oral and Maxillofacial Surgery, School of Dental Medicine, Universitat Internacional de Catalunya, Carrer de Josep Trueta, Sant Cugat del Vallès, 08195 Barcelona, Spain; 4 Department of Periodontology, School of Dental Medicine, University of Bern, Freiburgstrasse 7, 3010 Bern, Switzerland; 5 Faculty of Health Sciences, University of Zaragoza, C/ Domingo Miral S/N, 50009 Zaragoza, Zaragoza Spain; 6 Fundació Institut Universitari per a la Recerca a l’Atenció Primària de Salut Jordi Gol i Gurina, Barcelona, Spain

**Keywords:** Health care, Medical research

## Abstract

Capacitive–resistive energy transfer therapy (CRet) is used to improve the rehabilitation of different injuries. This study aimed to evaluate and compare the changes in temperature and current flow during different CRet applications on upper and lower molars and incisors, with and without implants, on ten cryopreserved corpses. Temperatures were taken on molars and incisors with invasive devices and skin temperature was taken with a digital thermometer at the beginning and after treatments. Four interventions: 15 VA capacitive hypothermic (CAPH), 8 watts resistive (RES8), 20 watts resistive (RES20) and 75 VA capacitive (CAP75) were performed for 5 min each. All treatments in this study generated current flow (more than 0.00005 A/m^2^) and did not generate a significant temperature increase (p > 0.05). However, RES20 application slightly increased surface temperature on incisors without implants (p = 0.010), and molar with (p = 0.001) and without implant (p = 0.008). Also, CAP75 application increased surface temperature on molars with implant (p = 0.002) and upper incisor with implant (p = 0.001). In conclusion, RES8 and CAPH applications seem to be the best options to achieve current flow without an increase in temperature on molars and incisors with and without implants.

## Introduction

Dental implants are the most commonly used procedure to replace missing teeth^1^. A Dental implant consists of a piece of metal, usually titanium or titanium alloys, inserted and integrated into the bone, giving solid support for the final dental prosthesis. It is estimated that 12–18 million implants are inserted worldwide every year^2^. Furthermore, it is supposed to be one of the most common surgeries in the health field. Successfully integrated implants have a survival rate of more than 96.7% after 8 years^3^. Nowadays, the insertion of a dental implant requires a low-invasive surgical technique^4^. However, inflammation during the healing phase may be present, and the use of anti-inflammatory drugs (ibuprofen, indomethacin, diclofenac or celecoxib) are usually prescribed^5^.

Inflammation is a naturally occurring event following the early stages of tissue healing after an injury, or in this case, after a dental implant procedure. Ensuring a rapid and short inflammatory phase guarantees an early start of the proliferative phase. The invasion and proliferation of mesenchymal and endothelial (cells or tissue) will create the appropriate network for the dental implant osteointegration^6^. An excessive inflammatory reaction can cause the rejection of the implanted device^7–9^. This exacerbated inflammation can be multifactorial; some conditions or attitudes that conduce to rejection of implanted devices are: an inadequate surgical protocol, increased temperature of the bone bed, contamination of the surgical site and/or implanted device or an allergic adverse reaction^7–9^. After inflammation, the proliferative phase occurs, and new tissue grows to close the wound^6^. Any protocol or action towards increasing the speed of cell proliferation or migration results in favor of a successful osseointegration of dental implants. For this reason, capacitive-resistive energy transfer (CRet) can help increase the speed of cell proliferation and healing^10^. This technique is commonly used to treat muscular, bone, joint and tendinous lesions in the sports traumatology field^11–14^. CRet is a non-invasive electrothermic therapy classified as deep thermotherapy. Which consists of applying an electric current in the radiofrequency range of 300 kHz–1.2 MHz^15^. CRet therapy can be applied in two different treatment modes: capacitive and resistive. Capacitive mode is provided with an insulating ceramic layer and the energetic transmission generates heat in superficial tissue layers, with a selective action in tissues with low-impedance (water-rich)^16^. The resistive mode has no insulating ceramic layer; the radiofrequency energy passes directly through the body in the direction of the inactive electrode, generating heat in the deeper and more resistant tissues (with less water content)^16^.

The physiological effects of CRet therapy are generated by the application of an electromagnetic field, with a frequency of approximately 0.5 MHz, to the human body. The effects attributed to this technique include increased blood circulation, lymphatic effects, increased cell proliferation and, if desired, a thermal increase of deep and superficial structures^15,17,18^. Some of these reactions, such as increased blood perfusion, are related to an increase in temperature in the tissue. However, others, such as cell proliferation, appear to be more related to the flow of electric current^17^. It has been described that at 0.00005 A/m^2^ of current flow, the phenomenon of cell proliferation begins^19^.

Currently, there are five studies analyzing thermal changes and current flow in cadavers using CRet therapy^15,16,18,20,21^. These studies focus on the Achilles tendon region and the myotendinous junction of the gastrocnemius muscles^15^, on the capsular and intra-articular structures of the knee^18^, on the tendon and capsular structures of the glenohumeral joint^20^, on a clinical protocol for the elbow region^21^ and biceps femoris and quadriceps^16^. However, no study has been found that assesses changes in temperature and current flow in intraoral structures such as teeth or dental implants. The relevance of generating current flow and cell proliferation (cell proliferation stimulated by current flow) without excessively increasing or even decreasing the temperature could set a precedent for a more effective recovery of acute dental implant patients during their consequent inflammatory phase. It is unknown whether the presence of an implant can generate a higher temperature increase. For this reason, it seems interesting to study the differences in order to be able to perform safe treatments in living patients.

The objective was to evaluate and compare the changes in temperature and current flow of different CRet applications on upper and lower molars and incisors with and without implants by performing invasive measurements on cadaveric specimens.

The null hypothesis was that there are no significant temperature differences in the application of CRet therapy in cadaveric specimens with and without implants and that there is no current flow in cadaveric specimens.

The alternative hypothesis was that there are significant temperature differences in the application of CRet therapy in cadaveric samples with and without implants and that there is current flow in the cadaveric samples.

## Results

The current flow was stable during the applications. The incisor region without implants showed values of 0.12 A ± 0.1 (CAPH), 0.23 A ± 0.2 (CAP75), 0.24 A ± 0.1 (RES8) and 0.36 A ± 0.2 (RES20). In the incisor region with implants, we found values of 0.24 A ± 0.14 (CAPH), 0.29 A ± 0.22 (CAP75), 0.27 A ± 0.09 (RES8) and 0.47 A ± 0.15 (RES20).

In the molar region without implants, we found values of 0.09 A ± 0.1 (CAPH), 0.18 A ± 0.19 (CAP75), 0.18 A ± 0.15 (RES8) and 0.27 A ± 0.24 (RES20). In the molar region with implants, values of 0.13 A ± 0.02 (CAPH), 0.26 A ± 0.03 (CAP75), 0.31 A ± 0.05 (RES8) and 0.47 A ± 0.08 (RES20) were found.

The temperature recorded during the different applications at the beginning and at the end of the treatment, both in the superficial region and in the upper and lower molar/incisor region, is shown in Table 1 for the incisors and in Table 2 for the molars.

**Table 1:** Incisor with and without implants.

		Baseline	End of treatment	Difference (95% CI)	Within-group p
Superficial	CAPH—implants	19.32 ± 1.38	20.06 ± 1.76	0.74 [− 0.34, 1.82]	0.548
	CAPH—no implants	18.80 ± 2.23	19.04 ± 1.92	0.24 [− 0.84, 1.32]	1.000
				Between-group p = 0.262 F = 1.342	
	CAP75—implants	19.52 ± 1.47	20.09 ± 2.24	0.57 [− 1.51, 2.65]	1.000
	CAP75—no implants	19.70 ± 2.16	21.31 ± 3.73	1.61 [− 0.47, 3.69]	0.290
				Between-group p = 0.229 F = 1.553	
	RES8—implants	18.33 ± 1.38	19.06 ± 1.49	0.73 [− 0.35, 1.81]	0.598
	RES8—no implants	18.60 ± 1.92	19.24 ± 2.27	0.64 [− 0.44, 1.72]	1.000
				Between-group p = 0.838 F = 0.043	
	RES20—implants	18.49 ± 1.24	18.66 ± 1.16	0.17 [− 1.34, 1.68]	1.000
	RES20—no Implants	17.89 ± 2.28	19.70 ± 3.09	1.81 [0.30, 3.32]	0.010
				Between-group p = 0.014 F = 7.369	
Upper	CAPH—implants	25.94 ± 3.56	27.53 ± 4.81	1.59 [− 0.47, 3.65]	0.289
	CAPH—no implants	24.98 ± 3.79	25.14 ± 3.75	0.16 [− 1.90, 2.22]	1.000
				Between-group p = 0.100 F = 3.012	
	CAP75—implants	25.59 ± 4.18	27.59 ± 3.94	2.00 [0.83, 3.17]	< 0.001
	CAP75—no implants	25.16 ± 4.04	25.87 ± 4.46	0.71 [− 0.46, 1.88]	0.968
				Between-group p = 0.013 F = 7.576	
	RES8—implants	30.02 ± 2.26	31.43 ± 1.73	1.41 [− 0.54, 3.36]	0.417
	RES8—no implants	25.53 ± 4.42	26.41 ± 4.80	0.88 [− 1.07, 2.83]	1.000
				Between-group p = 0.506 F = 0.461	
	RES20—implants	30.57 ± 3.28	31.35 ± 2.06	0.78 [− 1.10, 2.66]	1.000
	RES20—no implants	23.81 ± 5.52	26.21 ± 4.87	2.40 [0.52, 4.28]	0.006
				Between-group p = 0.045 F = 4.654	
Lower	CAPH—implants	22.60 ± 2.08	24.77 ± 1.84	2.17 [− 0.05, 4.39]	0.059
	CAPH—no implants	21.00 ± 3.28	20.61 ± 4.10	− 0.39 [− 2.61, 1.83]	1.000
				Between-group p = 0.010 F = 8.320	
	CAP75—implants	21.74 ± 3.06	22.78 ± 2.89	1.04 [− 1.04, 3.12]	1.000
	CAP75—no implants	20.94 ± 4.37	20.62 ± 5.45	− 0.32 [− 2.40, 1.76]	1.000
				Between-group p = 0.121 F = 2.653	
	RES8—implants	24.73 ± 2.51	25.88 ± 2.30	1.15 [− 0.90, 3.20]	1.000
	RES8—no implants	20.74 ± 3.79	21.07 ± 5.03	0.33 [− 1.72, 2.38]	1.000
				Between-group p = 0.331 F = 0.997	
	RES20—implants	24.78 ± 2.55	24.80 ± 3.00	0.02 [− 3.05, 3.09]	1.000
	RES20—no implants	19.71 ± 6.05	20.56 ± 5.31	0.85 [− 2.22, 3.92]	1.000
				Between-group p = 0.508 F = 0.455	

*CI* confidence interval, *CAPH* capacitive hypothermic, *CAP75*: capacitive 75 VA, *RES8* resistive 8 watts, *RES20* resistive 20 watts.

**Table 2:** Molar with and without implants.

		Baseline	End of treatment	Difference (95% CI)	Within-group p
Superficial	CAPH—implants	17.76 ± 1.95	19.08 ± 1.43	1.32 [− 0.24, 2.88]	0.163
	CAPH—no implants	19.54 ± 2.48	19.97 ± 2.66	0.43 [− 1.13, 1.99]	1.000
				Between-group p = 0.170 F = 2.041	
	CAP75—implants	18.26 ± 1.80	24.67 ± 3.04	6.41 [1.94, 10.88]	0.002
	CAP75—no implants	20.25 ± 3.01	24.95 ± 6.01	4.70 [0.23, 9.17]	0.033
				Between-group p = 0.352 F = 0.915	
	RES8—implants	17.63 ± 1.56	19.37 ± 1.44	1.74 [− 0.13, 3.61]	0.086
	RES8—no implants	18.76 ± 1.90	20.57 ± 3.69	1.81 [− 0.61, 3.68]	0.064
				Between-group p = 0.927 F = 0.009	
	RES20—implants	18.32 ± 1.56	22.73 ± 2.10	4.41 [1.73, 7.09]	< 0.001
	RES20—no implants	19.16 ± 3.58	22.48 ± 5.29	3.32 [0.64, 6.00]	0.008
				Between-group p = 0.323 F = 1.032	
Upper	CAPH—implants	26.85 ± 3,64	27.16 ± 3.72	0.31 [− 1.24, 1.86]	1.000
	CAPH—no implants	23.78 ± 4,99	23.33 ± 5.54	-0.45 [-2.00, 1.10]	1.000
				Between-group p = 0.237 F = 1.497	
	CAP75—implants	26.59 ± 3.90	26.89 ± 5.39	0.30 [− 2.68, 3.28]	1.000
	CAP75—no implants	24.17 ± 4.85	24.17 ± 6.19	0.00 [− 2.98, 2.98]	1.000
				Between-group p = 0.804 F = 0.063	
	RES8—Implants	27.60 ± 5.16	28.47 ± 4.15	0.87 [− 0.90, 2.64]	1.000
	RES8—No Implants	24.57 ± 4.85	24.47 ± 6.31	− 0.10 [− 1.87, 1.67]	1.000
				Between-group p = 0.188 F = 1.873	
	RES20—implants	26.50 ± 4.61	27.40 ± 4.05	0.90 [− 0.84, 2.64]	1.000
	RES20—no implants	23.51 ± 5.60	25.05 ± 5.95	1.54 [− 0.20, 3.28]	0.121
				Between-group p = 0.370 F = 0.846	
Lower	CAPH—implants	23.11 ± 3.03	23.57 ± 3.00	0.46 [− 0.75, 1.67]	1.000
	CAPH—no implants	21.54 ± 4.54	20.84 ± 4.41	− 0.70 [− 1.91, 0.51]	1.000
				Between-group p = 0.028 F = 5.695	
	CAP75—implants	23.32 ± 3.12	24.69 ± 3.19	1.37 [− 0.95, 3.69]	1.000
	CAP75—no implants	20.48 ± 5.15	20.53 ± 5.36	0.05 [− 2.27, 2.37]	1.000
				Between-group p = 0.173 F = 2.011	
	RES8—implants	24.19 ± 2.51	23.87 ± 2.50	− 0.32 [− 2.13, 1.49]	1.000
	RES8—no implants	20.93 ± 4.75	21.26 ± 5.09	0.33 [− 1.48, 2.14]	1.000
				Between-group p = 0.383 F = 0.800	
	RES20—implants	23.93 ± 2.53	23.64 ± 2.87	− 0.29 [− 3.69, 3.05]	1.000
	RES20—no implants	20.04 ± 6.48	20.22 ± 6.06	0.18 [− 3.16, 3.52]	1.000
				Between-group p = 0.729 F = 0.124	

*CI* confidence interval, *CAPH* capacitive hypothermic, *CAP75* capacitive 75 VA, *RES8* resistive 8 watts, *RES20* Resistive 20 watts.

In the between-group analysis of the incisors, statistically significant differences were found in the superficial region temperature of the RES20 application (p = 0.014), the upper incisors temperature of the CAP75 application (p = 0.013) and RES20 (p = 0.045), in the lower incisor’s temperature of the CAPH application (p = 0.010). In the within-group analysis of the incisors, statistically significant differences were found in the RES20 application in the superficial region temperature (p = 0.010) and in the upper incisor’s temperature (p = 0.006) in the non-implant group with an increase in temperature of 1.81° and 2.40° respectively.

In the between-group analysis of molars only, statistically significant differences were found in the lower molar temperature of the CAPH application (p = 0.028). In the within-group analysis of the molars, statistically significant differences were found in the superficial region temperature of the CAP75 and RES20 applications. In the CAP75 application, the group with implants (p = 0.002) and without implants (p = 0.033) showed a superficial temperature increase of 6.41° and 4.70°, respectively. In the RES20 application, the group with implants (p < 0.001) and without implants (p = 0.008) showed a superficial temperature increase of 4.41° and 3.32°, respectively.

## Discussion

Based on the results obtained, the alternative hypothesis, that there are significant temperature differences in the application of CRet therapy in the cadaveric samples with and without implants and that there is current flow in the cadaveric samples, can be accepted. Therefore, the null hypothesis, that there are no significant temperature differences in the application of CRet therapy in cadaveric specimens with and without implants and that there is no current flow in cadaveric specimens, can be rejected. However, in two applications (RES8 and CAPH) no significant temperature differences were observed between the implant and non-implant groups.

The highest significant temperature rise produced in the upper and lower molar and incisor applications was only 2.4° of temperature at the surface level. In addition, the living tissue would likely experience an even lower temperature rise since, in these subjects, the blood actively circulates through the body dissipating heat to adjacent areas^22^. This process, called thermoregulation, prevents unwanted hyperthermia in nearby tissues, as well as excessive heat during treatment^22^.

As with any implantable device in hard tissues, dental implants need to be osseointegrated in the bone to be functional^23^. Osseointegration is an ordered cascade of events that lead to the integration of an implantable device into a hard-living tissue. Osseointegration follows the sequence of tissue healing, being among the first steps in the inflammatory phase. In a physiological environment, the inflammatory phase begins 10 min after the injury and takes hours to days to end^24^; a shorter time is considered better for the healing process. After the injury, platelets arrive, and first events occur, such as degranulation and histamine derived vasodilatation, leading to increased blood flow and decreased stream velocity. Limiting the inflammatory phase, we ensure to move as quickly as possible to the proliferative phase. A recent publication studying the effects of CRet on cell proliferation and migration showed that 6 h after the application, migration and proliferation were increased. In this same study, results after 12 h of the CRet application showed a slight decrease in the phosphorylation of p38 proteins and a slight increase of other proteins related to a MAPK pathway, possibly indicating that the CRet treatments might have an anti-inflammatory effect (as well as a proliferative induction/effect)^10^. Achieving cellular proliferation could be indicated in inflammatory pathologies that need tissue regeneration, especially during the first 2 weeks^25,26^.

The evolution from the inflammatory to the proliferative phase encompasses the formation of a new collagenous extracellular matrix and angiogenesis. The duration of this phase lasts from a few days to a few weeks. Fibroblasts will start to proliferate via integrins and migrate using an amoeboid movement from the healthy surrounding tissue into the wound, where the blood clot is forming, and inflammatory cells are present^27^. The proliferative phase also includes the formation of new blood vessels in the wound site, which is a mandatory prerequisite for tissue healing^27^.

In this way, CRet therapy could be a strategy to increase cell proliferation without increasing the temperature (which would be negative in an inflammatory phase of the tissues)^19^. This improvement in cell proliferation would result in less pain for patients and possible better consolidation of the implant in a shorter time^15,17,18^. However, this hypothesis should be verified in future studies with living patients.

This study has several limitations, which are described below. First, it is a study performed on cadavers in which there is no thermoregulation or active blood circulation. In living subjects, the body's thermoregulatory effect dissipates heat, probably producing more significant temperature increases in the cadaveric samples than those expected in living subjects^22^. Another limitation is found in the properties of the tissues. Another limitation is that cadaveric studies are performed with a small sample size, and despite being cryopreserved cadavers, the properties of the tissues may vary slightly from those of living subjects. Despite these limitations, the authors consider that the use of donor bodies has made it possible to know how different CRet applications affect the temperature and current flow values in the molar and incisor region and to know their effects and applicability before applying them to real patients.

In conclusion, all applications used in this study generated current flow and did not generate significant temperature increases in the tooth region with or without implants. However, the RES20 and CAP75 applications generated a thermal increase in some conditions. So, RES8 and CAPH applications seem to be the best options to achieve current flow without temperature increase in molars and incisors with and without implants. These basic science results may be the precedent for using RES8 and CAPH applications in living patients.

## Methods

### Study design

A cross-sectional study was conducted to determine the effect of electrical resistive/capacitive energy transfer of the T-Plus (Wintecare SA, Chiasso, Switzerland) device on temperature and current in the intraoral region (incisors and molars with and without implants) in cadaveric samples. The body donation program of the Faculty of Medicine and Health Sciences of the Universitat Internacional de Catalunya provided all the samples. This study was approved by the ethical committee “Comitè d’Ètica de Recerca (CER) of Universitat Internacional de Catalunya” with reference number CBAS-2019-17 approved on April 4, 2022.

### Cadaveric sample

The sample consisted of 10 complete corpses (5 with implants and 5 without implants), cryopreserved and fresh. Measurements were taken on the right and left molars and the right and left incisors on each corpse. This generated a total sample of 10 molars with implants, 10 incisors with implants, 10 molars without implants and 10 incisors without implants. The cadavers were stored at 3 °C and kept at room temperature^18^ for 36 h before the study^16^. The mean age of the cadavers was 67.7 [± 6.0] years. None of the cadaveric specimens used for this study had evidence of trauma or surgical scars in the craniomandibular region.

### Intervention

**Figure 1:**
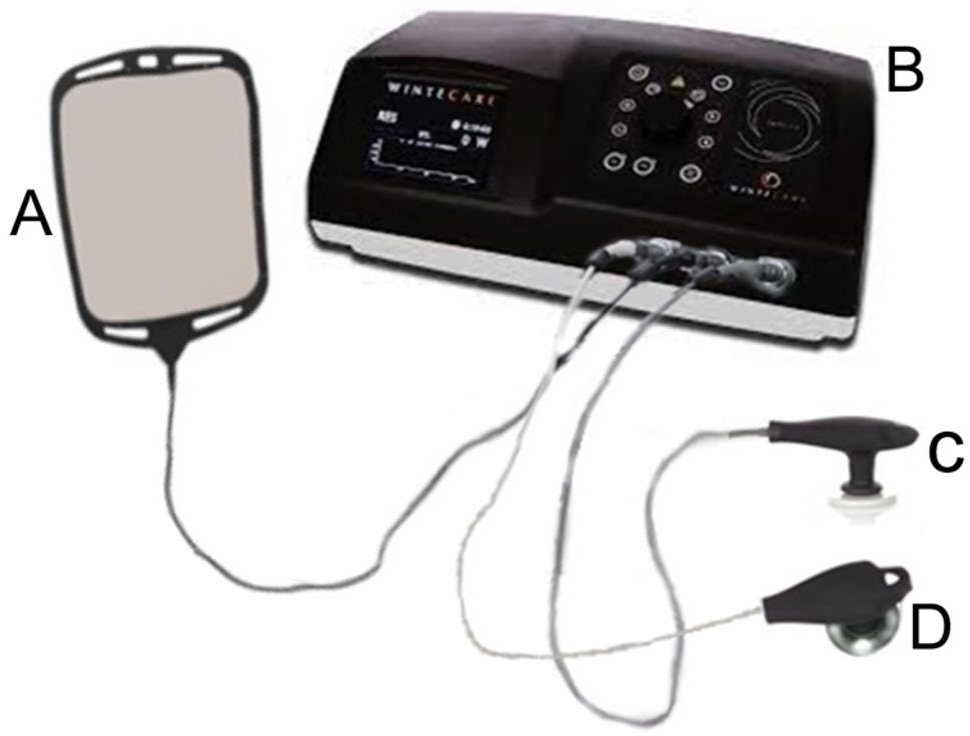
Schematic of the CRet device. (A) Return electrode (base plate); (B) T-Plus Control Center; (C) capacitive electrode; (D) resistive electrode.

The power range of a T-Plus (Wintecare SA, Chiasso, Switzerland) (Fig. 1) device used in this study varies from 1 to 300 watts in resistive mode and from 1 to 450 VA in capacitive mode^15^. The power was determined by the protocol used depending on the region to be treated. In the mandibular region, the aim was not to generate an undesired increase in temperature, knowing that an increase in temperature could lead to an increase in inflammation and thus a rejection of the dental implant^4^.

In this study, low power applications were used, not exceeding the 0.3 A limit^15^. Applications were performed with a 15 VA capacitive hypothermic electrode (CAPH), 8 watts resistive (RES8), 20 watts resistive (RES20) and 75 VA capacitive (CAP75).

**Figure 2:**
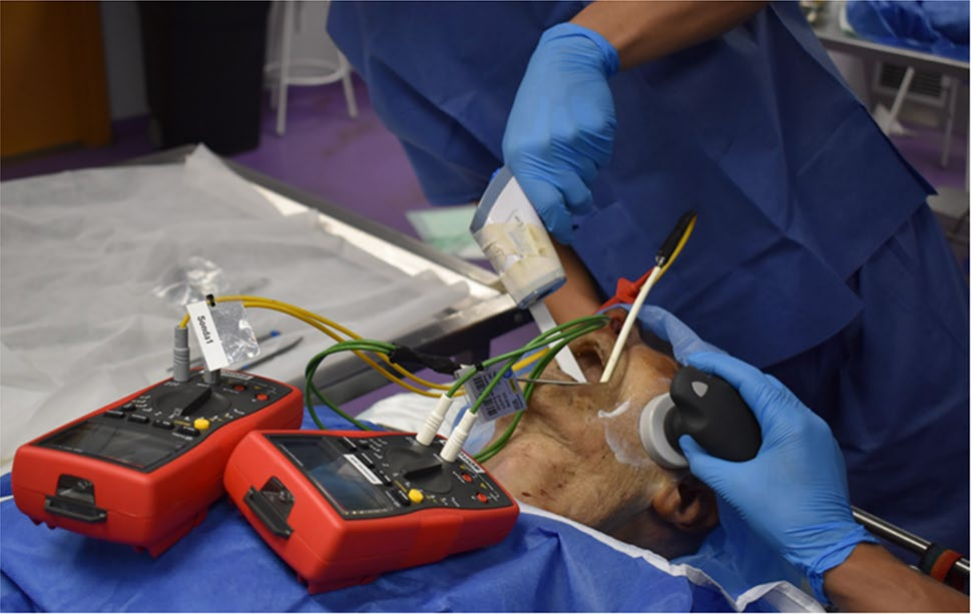
Example of application of CRet intervention (CAPH) in molars and placement of temperature gauges.

Four interventions were performed (CAPH, RES8, RES20 and CAP75) for 5 min each. The base plate was placed in the scapular area of the specimen contralateral to the side to be treated. The mobile electrode was placed in the lateral region of the jaws for treatment on the molars and the anterior area for the incisors (Fig. 2). Dynamic movements like those used with real patients were performed with constant pressure. The treatments were performed by a physiotherapist (HGC) with more than 10 years of experience in the use of T-Plus.

### Experimental procedure

Each cadaver was placed in supine position with partially opened mouth.

A dentist (CSJ) with 10 years of experience performed the extraction of teeth from the cadavers and the placement of implants in the implicated specimens (Bone level tapered implants, 4.1 mm × 13 mm (diameter × length); Roxolid, Basel, Switzerland) in the incisors and molars. In the case of the samples without implants, only tooth extraction was performed to measure the implant's exact point.

The order of the treatment protocols and the treatment of the cadavers were randomized prior to the study. An external researcher carried out this randomization using the “random.org” software. Before applying each treatment it was ensured that the basal temperature of each corpse returned to the initial values before applying the next treatment.

Before starting the measurements it was ensured that all the instrumentation used had a calibration certificate. Invasive temperature devices “Hart Scientific PT25 5628-15 (Fluke, Everett, Washington, USA)” were used to measure the temperature (°C) of the molars and incisors. One of the gauges was placed on the lower molar/incisor and the other on the upper molar/incisor of the same side to be treated. In the case of the implant group, this gauge was placed in contact with the implant. In the case of subjects without implants, the temperature gauge was placed right in the tooth socket. A “Thermocomed (AB Medica Group, Barcelona, Spain)” digital thermometer was used to measure the superficial temperature of the skin in the mandibular region.

The T-Plus’s return electrode (base plate) was placed on the scapular area of the cadavers. The treatment was performed with the mobile electrode of the T-Plus in the previously explained treatment region according to each application for 5 min. Initial surface and deep tissue temperatures were measured. These measurements were recorded before starting the application and immediately after finishing (at the end of the 5-min treatment). In addition, temperature changes during each minute of treatment were recorded as a temperature control measure. Before the treatment, impedance was always recorded (Multimeter Fluke 8846A) to ensure that the values marked by the T-Plus Wintecare device were correct. In addition, the actual current flow for each application was calculated using the average voltage divided by the initial impedance.

### Statistical analysis

Statistical analysis was performed with SPSS (Version 22.0; IBM, Armonk, NY, USA). Normal distribution was calculated with the Shapiro–Wilk test (p > 0.05). The mean and standard deviation of the superficial temperature and of the temperatures taken with the invasive devices were calculated depending on each application. For within-group analysis, the repeated samples ANOVA (2 × 4) test with Bonferroni’s post-hoc was used. For between-group analysis, the one-factor ANOVA test was used. The value of p < 0.05 was considered statistically significant.

### Ethics approval

The Comité d´Ètica de Recerca from Universitat Internacional de Catalunya approved the study (CBAS-2019-17) approved on April 4, 2022. The investigation conformed with the principles outlined in the Declaration of Helsinki. The informed consent from “body donors” was obtained before the death and any personal data was hidden.

## Data Availability

The datasets used and/or analyzed during the current study are available from the corresponding author on reasonable request.

## Abbreviations

CRet: Capacitive–resistive electric transfer
CAPH: 15 VA capacitive hypothermic electrode
RES8: 8 Watts resistive
RES20: 20 Watts resistive
CAP75: 75 VA capacitive
